# Translation, reliability, and validation of the Dutch Safe Use of Mobility Aid Checklist (SUMAC-NL) for walker use in people living with dementia

**DOI:** 10.12688/f1000research.132762.3

**Published:** 2025-06-30

**Authors:** Jesper Kroesen, Hans Hobbelen, Susan Hunter, Helen Bruinsma, Hans Drenth

**Affiliations:** 1 Physical Therapy Sciences, Universiteit Utrecht, Utrecht, Utrecht, The Netherlands; 2 FAITH research, Groningen/Leeuwarden, The Netherlands; 3 Research Group Healthy Ageing, Allied Health Care and Nursing, Hanzehogeschool Groningen, Groningen, Groningen, The Netherlands; 4 Department of General Practice & Elderly Care Medicine, Rijksuniversiteit Groningen, Groningen, Groningen, The Netherlands; 5 School of Physical Therapy, Faculty of Health Sciences, University of Western Ontario, Ontario, Ontario, Canada; 6 Treant, Emmen, 7285, The Netherlands; 7 ZuidOostZorg, Drachten, Friesland, The Netherlands

**Keywords:** dementia, walker, geriatric evaluation, fall risk, walking, translation, validity, reliability

## Abstract

**Background:**

People with dementia have a yearly risk of falling of 60 to 80 percent. Therefore, a walker is often recommended. However, the use of a walker in people with dementia is associated with a threefold increased odds of falls compared to their healthy peers who walk with a walker. Assessing walking skills with a walker can improve the quality of advice on its use. Therefore, a tool to assess functional walking skills with a walker is needed. The SUMAC was developed to fill this gap. So far, there is no Dutch instrument available that can assess functional walking skills with a walker in people with dementia.

**Methods:**

Reliability was evaluated by scoring videos of people with dementia (n = 9) using a walker performing the SUMAC-NL. ICC was used to assess inter-rater and test-retest reliability. An expert panel (n = 8) evaluated the content validity using the content validity index (CVI) and the content validity ratio (CVR).

**Results:**

Inter-rater reliability of the SUMAC-NL was statistically significant for the physical function (PF) domain (ICC = 0.94, 95%CI (0.84, 0.98, p < 0.001) and for the interaction with equipment (EQ) domain (ICC = 0.79, 95%CI (0.49 – 0.95), p < 0.001). Test-retest reliability was statistically significant for both the PF domain (ICC = 0.95, 95%CI (0.89, 0.99), p < 0.001) and EQ domain (ICC = 0.92, 95%CI (0.82, 0.98), p < 0.001). The SUMAC-NL shows content validity with a CVI >0.79 for both domains and a CVR of 0.53 on the PF domain and 0.78 on the EQ domain.

**Conclusions:**

The SUMAC-NL shows good to excellent reliability and content validity for both the PF and the EQ domain. The SUMAC-NL seems to be a promising tool to assess walking with a walker in people with dementia in the Netherlands.

## Introduction

Dementia is a neurocognitive disorder in which mental abilities as associative memory, reasoning and perceptual speed are severely reduced. ^1^ It is estimated that there are approximately 290,000 people with dementia in the Netherlands and it is the fastest-growing cause of death in the Netherlands. ^1^^–^^3^ Dementia is characterized by a cognitive decline related to memory and in at least one of the other domains: perceptual-motor function, language, executive function, attention, and social cognition. ^4^^,^^5^

People with dementia have a high risk of falling and the cause is multifactorial. Intrinsic and extrinsic factors such as vision or balance problems and the use of medication can play a role in falling. ^6^ Also, impairments in attention and executive functions are prominent risk factors for falls among people living with dementia. ^7^ Importantly, balance and gait problems are common and progressive in dementia as well. People with dementia have a yearly risk to fall of sixty to eighty percent. This is twice as high as in their cognitively healthy peers. ^7^^,^^8^ Moreover, people with dementia have a high risk of admission to a long-care setting. ^7^^,^^8^

To compensate for deficits in balance, gait and strength that accompany dementia, the use of a walker is often recommended. A walker is known for improving stability during walking in cognitively healthy adults. ^9^^,^^10^ However, the use of a walker in people with dementia is associated with a threefold increased odds of falls compared to their healthy peers who walk with a walker. ^6^^,^^11^ Lindemann *et al.* identified obstacles and opening a door towards a person as the main problems for older adults using a walker. ^12^

Many standardized scales for balance and gait developed for cognitively healthy older adults have been evaluated for reliability and validity in people living with dementia, such as the Step Test, the 6-minute walk test, Timed Up And Go Test (TUGT) and the Berg Balance Scale (BBS). ^13^ These existing measures provide only partial information regarding mobility and few target elements that become relevant as dementia progresses. ^13^ In addition, none of these instruments can assess the functional skills for safe walking when using a walker in people with dementia.

The high frequency of falls among people with dementia and the absence of a measurement instrument to assess walking skills in walker use led Hunter *et al.* to develop the Safe Use of Mobility Aid Checklist (SUMAC) in 2020. ^14^ The SUMAC was generated by a focus group (N = 12) consisting of one geriatrician, two registered nurses, five physiotherapists, and four occupational therapists. ^14^ The generated tasks were scored for relevance by an independent panel of five healthcare professionals. This resulted in a final selection of nine tasks in two domains (physical functioning (PF) and interaction with equipment (EQ) that form the SUMAC. The SUMAC can evaluate the skills of walking with a walker in people with dementia who walk with a walker independently or assess the success of training when the walker has been identified as an appropriate level of gait aid to compensate for deficits. ^14^ The SUMAC has a good test-retest and inter-rater reliability (ICC = 0.89 for PF and ICC = 0.88 for EQ) and shows good construct validity (r _s_ = 0.92 for the PF score and r _s_ = 0.82 for the EQ score). ^14^ This makes it a promising tool to give specific advice and training in walker use and to reduce the risk of falling in people with dementia.

Currently, there is no Dutch assessment instrument that can assess walking skills in people with dementia who need a walker. Because the SUMAC consists of specific tasks that must be performed exactly as originally intended and, above all, the extensively described observation items must be correctly interpreted by an professional. This is best done in the own language. Therefore, a Dutch measuring instrument must be developed that can assess the safety of walking with a walker in people with dementia. This will lead to better advice and training regarding the use of a walker and could contribute to reducing the risk of falls in people with dementia in the Netherlands.

Differences in language, culture and healthcare settings can affect how assessors interpret and score tasks, and how patients perform tasks. Therefore, there is international consensus that cross-cultural adaptation should always be accompanied by a formal process of validation and reliability testing in the new context.

Because the SUMAC is introduced in another country with a different language the instrument must be translated and culturally adapted to maintain content validity and reliability. ^15^^,^^16^

Therefore, the primary objective of this study is to 1) translate the SUMAC from English to Dutch and 2) determine the inter-rater reliability, the test-retest reliability, and the content validity of the SUMAC-NL to assess walking skills in older people with dementia living in the Netherlands who walk with a walker.

## Methods

### Study design

This study has an observational clinimetric design and involved two phases. The first part of this study consisted of the translation of the English version of the SUMAC into Dutch (SUMAC-NL). In the second part of this study, the reliability and validity evaluation of the SUMAC-NL was performed. The second part of the study was a replication study of the work by Hunter *et al.* (2020). ^14^

### Dutch translation of the SUMAC

**Figure 1.**
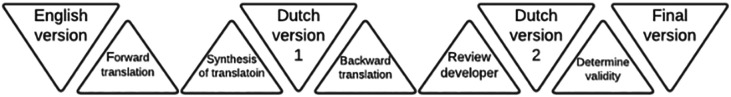
Translation process. ▽ = translated version; △ = step in translation.

The SUMAC was translated into Dutch between July and August 2021. The tasks and domains in the SUMAC remained the same. The translation was done according to the Beaton guideline ^15^ and consisted of five phases. The first phase was the forward translation from English to Dutch by two Dutch native speakers with a medical background. The first translator knew the purpose of the instrument. The second translator did not know the instrument to keep the translation as objective as possible. The second phase was the synthesis of the two translations by the Dutch native translators. The third phase was the backward translation from Dutch to English by two English native speakers. The first translator had a medical background and the second translator did not. Both translators were unaware of the purposes of the SUMAC. The fourth phase consisted of a review of the translations by the researcher, two experts, and the developer of the SUMAC. The fifth phase consisted of getting an understanding of the items. For the feasibility of this study, the last step has been replaced by the evaluation of the content validity ( Figure 1). This last step of the Beaton guideline consists of testing the prefinal version of the translated test. This involves administering the translated test to 30 to 40 people and then conducting interviews to ask participants what they thought was meant by the questions and the chosen response. ^15^

### Reliability and validity evaluation

*Participants*

Older people with dementia who walk with a walker, living in an intramural setting were recruited by their physiotherapist between 1 December 2021 and 28 February 2022 by convenience sampling. The recruiter selected potential participants from the institution’s patient database. Potential participants and their families were informed about the study through the information letter and through verbal explanations. They were then asked for their participation.

The inclusion criteria were: 1) diagnosed with dementia 2) walking with a walker, 3) Dutch-speaking, 4) able to follow simple instructions, 5) able to walk 60 meters without the supervision of another person, 6) able to perform the SUMAC-NL and 7) having someone available who can make decisions (e.g. on participation) if necessary. ^17^ Exclusion criteria for this study were: 1) comorbidities that limit mobility, such as Multiple Sclerosis (MS), a past Cerebro Vascular Accident (CVA), or (poly) neuropathy, and 2) people who have recently undergone surgery that limits mobility. All participants provided written informed consent. The legal representative of the participant was asked to give informed consent if the participant was deemed unable to do so.

*Data collection*

The demographic data requested from the participants were age, sex, amount of medication use, number of comorbidities, level of activities of daily living, and fear of falling. The 6-item Katz ADL scale was used to assess the participants’ ability to perform ADL activities and the short-FES-I is administered to determine the fear of falling of the participants. The Katz ADL (ICC = 0.82, *p* < 0.05) and short-FES-I (α = 0.84) are both reliable instruments to measure these parameters. ^18^^–^^20^

The assessment of the performed tasks by the participants was done by a group of raters consisting of a convenience sample of five physiotherapists who were recruited by one of the researchers (JK). All physiotherapists provided written informed consent for using their demographic data at the start of data collection. The physiotherapists were all legally registered therapists working in the physical therapy primary practice. This group of raters only participated in the reliability part of this study.

The participants were asked to perform the tasks of the SUMAC-NL while being filmed. Filming took place on 25 February and 4 March 2022 in a corridor with sufficient space in the institution where the participants live. Participants were asked if they wanted to be recognised in the video. If they did not agree, their faces were blurred in the videos so that the participants are unrecognisable in the video. The videos were stored in a 2FA-protected digital location, according to the data management plan and were deleted after the raters finished rating the videos. These videos were only used for the reliability assessment.

*Ethical considerations*

This study was conducted following the Declaration of Helsinki and the FAIR principle and was approved by the Hanze University of Applied Sciences Groningen’s Ethical Review Board (HEAC), with approval number heac.2021.026. Participants gave informed consent for understanding the information letters and that participating in the study was voluntary. In addition, they gave permission to contact their GP in case of unexpected findings, the collection and use of data and longer storage of the data as well as whether or not they wanted to be contacted again for follow-up studies.

*Reliability*

Each of the raters was asked to watch the provided instruction video and to read the instruction manual of the SUMAC-NL before starting the evaluation of the participants. The instruction video consisted of a detailed instruction of the components and items within the SUMAC-NL and an example of the evaluation with the SUMAC-NL. The videos of the participants performing the SUMAC-NL were sent to the raters on 11 March 2022. The evaluations of the participants was done twice at an interval of one week. The ratings were collected by the researcher on 8 April 2022.

The order of the tasks in the videos was the same as in the SUMAC-NL. The videos were offered in a random order. This order differed between the first and second evaluation session. Each rater evaluated all participants. The raters could evaluate the videos digitally at a location of their choice.

*Validity*

An expert panel was established to determine content validity. This group is composed of experts other than the reliability section raters. The expert panel consisted of six master of science trained geriatric physiotherapists, one master of science geriatric physiotherapist student, and one master of science trained physiotherapist. The experts in the panel have a mean age of 31.5 ± 2.7 years. Each of them has experience in working with older people with dementia (3.5 ± 3.2 years).

Before determining content validity, the expert panel received an instructional video explaining how to score the content of the SUMAC-NL. In addition, the expert panel received a digital score sheet in Microsoft Office Word in which the assessment of the SUMAC-NL was recorded. The scores of the experts were compared with each other and the content validity ratio (CVR) and content validity index (CVI) were calculated. Scoring of the SUMAC-NL by the expert panel to determine content validity took place between 18 February 2022 and 25 March 2025.

### Statistical analysis

IBM SPSS statistical package version 28 (IBM Corp, Armonk, NY) was used for descriptive analysis and reliability analysis. Microsoft Office Excel 2019 was used in the content validity calculation.

*Reliability*

An *a priori* sample size calculation for the reliability study was done using the formula of Shoukri *et al.* (2004). ^21^ This formula showed that a sample consisting of nine participants and five raters making two evaluations is required to determine the desired ICC of >0.9 (CI ± 0.1). An ICC of >0.90 is considered excellent reliability. ^22^ The demographic characteristics of the participants are presented using descriptive statistics.

Continuous data (SUMAC-NL subscores) were used and presented quantitatively. Missing data were considered ‘missing completely at random’ (MCAR) and imputed by the Expectation-Maximization (E-M) algorithm. ^23^ Normality checks were done by the Shapiro-Wilk test and using histograms and Q-Q plots.

The absolute reliability was calculated using the standard error of the measurement (SEM) and minimal detectable change with a 95% confidence interval (MDC _95_) for the PF and EQ domains of the SUMAC-NL. A smaller SEM means higher absolute reliability. ^24^ The SEM was estimated using the pooled standard deviation and the ICC for each group. The following formula was used: SE _measurement_ = *s* √(1- *r _xx_*). ^25^^,^^26^ The MDC _95_ was calculated using the SEM by the following formula: MDC _95_ = (1.96)*(√2)*(SEM). ^26^^,^^27^

The relative values of inter-rater and test-retest reliability were calculated using ICC. An average measurement, absolute agreement, 2-way random-effects model (ICC 2, *k*) was used for determining the ICC. ^22^ An ICC value less than 0.5 is indicative of poor reliability, values between 0.5 and 0.75 indicate moderate reliability, values between 0.75 and 0.9 indicate good reliability and values greater than 0.9 indicate excellent reliability. ^22^

*Content validity*

Content validity was determined using CVR and the CVI and was calculated separately for both domains of the SUMAC-NL. The CVR was used to show the acceptance of tasks in the instrument and the CVI was used to determine the content validity of both the entire domains. ^28^

For CVR, the expert panel separately scored each item of the SUMAC-NL on a three-point Likert scale where a score of one is essential, a score of two is useful but not essential and a score of three is not essential. After obtaining the scores of the assessments of the experts, the CVR was calculated based on the following formula in Microsoft Office Excel 2019, where n _e_ represents the number of panel members who have chosen the item of ‘essential’, and N is the number of panel members:

$$\mathrm{CVR}=\frac{n_{e}-\left(\frac{N}{2}\right)}{\frac{N}{2}}$$

The CVR varies between -1 and 1. A higher score indicates further agreement of members of the panel on the necessity of an item in the instrument. The CVR of each domain is compared with the values in Lawshe’s table, and if the CVR is equal to or higher than the value (0.75) in Lawshe’s table, the item is preserved. ^28^

To determine the CVI, all items of the instrument have been scored on three parameters by the experts: relevance, simplicity, and clarity. Each parameter was scored on a 4-point Likert scale from disagree to totally agree. The average of the total scores was taken from the table and the CVI of each item was calculated with the following formula:

$$\mathrm{CVI}=\frac{\left(\text{the number of experts giving}\;a\;\text{rating of either}\;3\;\text{or}\;4\right)}{\text{the number of experts}}$$

The CVI of both domains of the SUMAC-NL was determined by dividing the sum of the CVI scores per item by the number of items. ^29^^,^^30^ A CVI of >0.79 was considered appropriate. ^30^

## Results

Nine participants with dementia participated in this study. The sample consisted of five women (55.6%) and four men with a mean age of 85.56 (± 7.49). See Table 1 for the demographic characteristics of the participants.

**Table 1.** characteristics of people with dementia participating in reliability study (N = 9).

Variable	Mean ± SD, or n %
Age (years)	85.56 ± 7.49
Sex n (% female)	5 (55.6%)
FES-I score	12.11 ± 4.26
Katz ADL score	4.44 ± 2.00
Number of prescribed Medications	5.13 ± 1.13
Number of Comorbidities	3.33 ± 1.50

SD = standard deviation.

### Reliability

The absolute reliability values were: SEM for the PF domain was 1.20 and for the EQ domain was 2.22; the MDC _95_ was 3.33 for the PF domain and was 6.15 for the EQ domain ( Table 2).

**Table 2.** Reliability values and scores for the two categories of the SUMAC-NL.

Domains of the Dutch Safe Use of Mobility Aid Checklist		
	Physical functioning (PF)	Interaction with walker (EQ)
Mean (SD), range		
Assessment 1	26.75 (4.83), 18-37	41.83 (7.76), 23-56
Assessment 2	26.13 (4.98), 16-36	40.78 (7.03), 28-55
Intraclass correlation coefficients (95%CI), *p*-value		
Assessment 1: inter-rater reliability	0.85 (0.63, 0.96), *p* < 0.001	0.86 (0.64, 0.96), *p* < 0.001
Assessment 2: inter-rater reliability	0.90 (0.74, 0.97), *p* < 0.001	0.75 (0.41 – 0.94), *p* < 0.001
Test-retest reliability	0.94 (0.86, 0.99), *p* < 0.001	0.91 (0.79, 0.98), *p* < 0.001
Absolute reliability		
Standard Error of Measurement (SEM)	1.20	2.22
Minimal Detectable Change (MDC _95_)	3.33	6.15

SD = standard deviation, CI = confidence interval.

A good to excellent inter-rater reliability in both evaluation moments in the PF domain (ICC = 0.85 (0.63, 0.96), *p* < 0.001 for assessment 1 and ICC = 0.90 (0.74, 0.97), *p* < 0.001 for assessment 2) and EQ domain (ICC = 0.86 (0.64, 0.96), *p* < 0.001 for assessment 1 and ICC = 0.75 (0.41 – 0.94), *p* < 0.001 in assessment 2) was found ( Table 2). In addition, we found excellent test-retest reliability in the PF (ICC = 0.94, 95%CI (0.86, 0.99), *p* < 0.001) and EQ (ICC = 0.91 95%IC (0.79, 0.98), *p* < 0.001) domains ( Table 2). The full raw data can be found under *Underlying data.* ^31^

### Validity

The evaluation of the content validity was done by an expert panel (N =8) with experience in working with older people with dementia.

In the evaluation of the content validity, we calculated a CVI of 0.86 on the PF domain and 0.96 on the EQ domain. In addition, we calculated the CVR of 0.53 on the PF domain and 0.78 on the EQ domain ( Table 3).

**Table 3.** CVI and CVR of the SUMAC-NL.

Domains of the Dutch Safe Use of Mobility Aid Checklist		
	Physical functioning (PF)	Interaction with walker (EQ)
CVI	0.86	0.96
CVR	0.53	0.78

CVI = content validity index, CVR = content validity ratio.

The CVI of all individual tasks was >0.79. The CVR of tasks 4 and 5 in the PF domain were both 0, of task 7 it was 0.5, and of tasks 8 and 9, it was 0.25. The other tasks showed a CVR >0.75. In the EQ domain, task 4 had a CVR of 0 and task 5 had a CVR of 0.5. The other tasks had a CVR >0.75.

## Discussion

This replication study of the study from Hunter *et al.* ^14^ includes the translation of the English SUMAC into Dutch and the determination of the test-retest reliability, inter-rater reliability, and validity of the Dutch SUMAC (SUMAC-NL) for people with dementia who walk with a walker. This study found a good to excellent inter-rater reliability and test-retest reliability, and strong support for content validity.

Small differences were found between our results and the results of the study by Hunter *et al.* ^14^ The inter-rater reliability of the SUMAC-NL seems to be higher than the English version. The test-retest reliability of the SUMAC-NL seems higher for both the PF domain and the EQ domain (0.94 vs. 0.89 and 0.91 vs. 0.88, respectively). The SEM is smaller for the PF domain (1.20 vs. 1.31) and higher for the EQ domain (2.22 vs. 1.93) in the SUMAC-NL. The MDC _95_ for the PF domain of the SUMAC-NL is smaller than that of the English-language SUMAC (3.33 vs. 3.64) and greater for the EQ domain (6.15 vs. 5.35). These differences could be explained by the differences in the way the raters were instructed. In the study by Hunter *et al.,* ^14^ the raters received a one-hour one-on-one training session while, in this study, an online education via an instructional video was chosen due to the COVID-19 pandemic. ^14^ The content of the rater training for this study was not similar to the content of the training in the Hunter *et al.* (2020) study. ^14^ The explanation of how to administer the SUMAC-NL was provided via a video that was sent to the raters. The raters did not have the opportunity to ask questions if they were unclear, but it was indicated that there was room to ask questions afterwards. The raters didn’t ask any questions. Therefore, the researchers assumed that the method of administering the SUMAC-NL was clear. In the Hunter *et al.* (2020) study, ^14^ the raters had the opportunity to ask questions at any moment in the training due to the one-on-one training. As a result, the interpretation of the scoring method may differ slightly between the two raters’ groups because in the study by Hunter *et al.* ^14^ the raters could be directly adjusted and in this study the raters relied mainly on their own interpretation.

The content validity index (CVI) shows that both the PF domain and the EQ domain are considered valid by the expert panel. However, the content validity ratio (CVR) shows that tasks 4, 5, 7, 8, and 9 are not valid (CVR <0.75) tasks for determining physical function (PF domain) for walking with a walker in people with dementia. This finding is not in line with the results from the study by Hunter *et al.* ^14^ The content validity was determined by two sets of experts to include these tasks important for observing physical functioning. ^14^ In addition, it appears that walking through a doorway causes serious problems for older people who use a walker, which is observed in tasks 7, 8, and 9. ^12^ Because of the results from previous research and a good CVR (>0.75) for tasks 7, 8, and 9 in the EQ domain, it seems appropriate to keep these tasks in the FF domain of the SUMAC-NL. ^12^

The expert panel in this study determined tasks 4 and 5 as not valid for both the PF and EQ domain. These tasks involve walking with a double task (horizontal head turns) and a cognitive task. The expert panel scored lower for these tasks on the simplicity parameter within the CVI. One of the reasons given is that the wording of the tasks can be interpreted in different ways. However, different studies show that gait performance decreases and fall risk increases with increasing task complexity with the addition of a second cognitive task in community-dwelling adults or cognitively impaired older adults. ^32^^–^^35^ Therefore, it seems appropriate to observe these performances and keep tasks 4 and 5 in the SUMAC-NL but the formulation may have to be modified in the future.

Other measuring instruments can score balance and physical performance to interpret a risk of falls. A commonly used measuring instrument for evaluating physical performance and walking in older adults is the Berg Balance Scale (BBS). ^36^^,^^37^ The BBS measures the balance of participants through transfers and static postures, without a walker. The BBS is a reliable instrument for determining the risk of falling and has good reliability in older people with dementia to assess physical functioning. ^36^^,^^38^^,^^39^ This measuring instrument can have value in determining walking safety but is not specified for determining walking safety in older people with dementia who walk with a walker. The SUMAC-NL seems to be an appropriate tool to fill this gap because of its good reliability in people with dementia and the functional items that are included for walking with a walker. The SUMAC-NL provides professionals a standardised assessment tool to assess safe walking with a walker in people with dementia.

Previous research shows that people with dementia are well able to learn tasks both implicitly and explicitly, making possible therapy effective. ^40^^,^^41^ The SUMAC-NL could be performed in people living with dementia who already walk with a walker or in people who have received training in walking with a walker so that the walking skills and/or the success of the training can be evaluated.

### Strengths and limitations

The translation of the English-language SUMAC into Dutch was carried out systematically using the Beaton guideline. ^15^ This increases the comparability between different cultures. ^42^ For the feasibility of this study, the last step in the guideline has not been reviewed. We have overcome this by determining the content validity and reliability, which also includes the comprehensibility of the instrument.

Another strength of this study is that the experts involved in the validation assessment of the SUMAC-NL were all master trained physiotherapists who are experts in the field of physical activity who have experience in geriatrics and work with people living with dementia. ^43^

This study also has some limitations. For the power and feasibility of this study, the reliability phase of this study consisted of several assessors (N = 5) and nine participants. Although the sample size is appropriate to achieve power in the analysis, research with a larger sample size (n >50) is recommended. ^44^ After data collection, there was missing data (<10%). This data was assumed to be missing completely at random (MCAR). After imputation, we did calculations on the imputed data and the raw data. The results of both calculations were similar to each other.

The variation in age of the expert group for determining content validity is small. The heterogeneity of the expert group was not explicitly taken into account when recruiting the expert group. As a result, the expert group is homogeneous in terms of age and experience. This reduces the generalizability of the results of this study to older or more experienced therapists. It may be useful to have a comparison of the content validity results with therapists with more experience in order to calibrate the results.

This study was done in older people with dementia who were able to perform the SUMAC-NL. Therefore, the results cannot be generalised to people with dementia who may not be able to complete the SUMAC-NL. The people in our sample show no severe fear of falling on the FES-I and are largely independent in ADL following the Katz ADL scores. The results are therefore not generalizable to people with severe fear of falling and are more dependent in ADL. Further research is needed on the psychometric properties of the SUMAC-NL in people who are less likely to be able to perform the SUMAC-NL, have a greater fear of falling and are more dependent in ADL.

Participants were included based on the diagnosis of dementia by a specialist in geriatrics. However, this did not include the degree of dementia. These data were not available at the time of recruitment. This makes the SUMAC-NL difficult to specify according to the cognitive state of an individual with dementia. In retrospect, this could have been remedied by taking the Montreal Cognitive Assessment (MoCA). ^45^

### Implications for practice and research

The inter-rater reliability, test-retest reliability, and validity support the use of the SUMAC-NL in practice. The SUMAC-NL should be considered to assess the walking skills in people living with dementia who use a walker independently and/or to evaluate the training success of walking with a walker. With the help of the SUMAC-NL, more specific training, education, and advice can be given for walker use.

The results of this study show that the Dutch version of the SUMAC has similar psychometric properties to the original Canadian version. This suggests that the original instrument is robust, and resistant to cultural and linguistic variations, reinforcing its generalisability and applicability. This is important because using non-validated translations in clinical practice or research can lead to measurement errors, misinterpretations and ultimately suboptimal care.

In addition, this study fills a gap in the Dutch context. Until now, there has been no validated instrument specifically designed to observe the safe use of a rollator by people with dementia in Dutch contexts. As such, this study provides concrete tools for professionals working with people with dementia.

Future research must determine whether the use of the SUMAC-NL can improve the walking safety of older people with dementia who walk with a walker and thus can reduce the risk of falling in this population. Because of the lack of generalizability of this study to older people with dementia with more severe fear of falling and are more dependent in ADL, further research into the psychometric properties of the SUMAC-NL in people with dementia is advised.

Finally, this translation and validation also provides opportunities for international comparison and collaboration. By using standardised and validated instruments in different countries, it will be possible to compare data on a larger scale and collectively contribute to evidence-based practice in walking skills for people with dementia who walk with a walker.

## Conclusion

In this study, the English-language SUMAC was translated into the Dutch SUMAC-NL. The SUMAC-NL seems to have good to excellent inter-rater reliability, test-retest reliability, and content validity. The SUMAC-NL seems to be a promising instrument for the assessment of walking with a walker in older people with dementia. The SUMAC-NL can be further developed into an instrument that can assess whether or not a person with dementia should walk with a walker.

## Data Availability

DataverseNL: Safe Use of Mobility Aid Checklist (SUMAC) for walker use in people living with dementia. https://doi.org/10.34894/RUKZQP ^31^ This project contains the following underlying data: Dataset of the reliability study
- DatastetreliabilitySUMACNL_15-5-2022_raw (.cvs and.sav) - Syntax1reliabilitySUMAC (.sps) Dataset of the validity study
-
Validityworksheet_CVI_CVR-SUMAC-NL (.xlsx) This project contains the following extended data:
-
README_ENG_ SUMAC_NL_translation_relibility_validation 27-1-2023.pdf
- informatiebrief SUMAC-NL studie 2-12-2021.docx (participant information sheet) - Handleiding SUMAC-NL_final.docx (manual for using the SUMAC-NL) - SUMAC-NL_final.docx (The official SUMAC-NL tool for clinical use) - Dataset definitions.pdf - The English version of the SUMAC can be found via: http://www.mobility-in-aging-lab.ca/#publications Data are available under the terms of the Creative Commons Zero “No rights reserved” data waiver (CC0 1.0 Public domain dedication).
