# Supplementary material for: Translation, reliability, and validation of the Dutch Safe Use of Mobility Aid Checklist (SUMAC-NL) for walker use in people living with dementia
Source: F1000Res. 2025 Jun 30;12:1150. Originally published 2023 Sep 14. [Version 3] doi: 10.12688/f1000research.132762.3 (PMC12284472; doi:10.12688/f1000research.132762.3)
Supplement: Peer review history

# Reviewer report: Reviewer response for version 3

**DOI:** 10.5256/f1000research.184144.r395676

**Published:** 2025-07-22

**Authors:** Jodie Marquez

**Affiliations:** 1 The University of Newcastle, Callaghan, New South Wales, Australia

Thank you to the editorial team for this robust and transparent process. 

 Much gratitude to the authors for their respectful and considered responses. I have no further comments and am pleased with indexing.

Is the work clearly and accurately presented and does it cite the current literature?

Partly

If applicable, is the statistical analysis and its interpretation appropriate?

Yes

Are all the source data underlying the results available to ensure full reproducibility?

Yes

Is the study design appropriate and is the work technically sound?

Yes

Are the conclusions drawn adequately supported by the results?

Partly

Are sufficient details of methods and analysis provided to allow replication by others?

Yes

Reviewer Expertise:

healthy aging, stroke, rehabilitation, falls

I confirm that I have read this submission and believe that I have an appropriate level of expertise to confirm that it is of an acceptable scientific standard.

# Reviewer report: Reviewer response for version 2

**DOI:** 10.5256/f1000research.180410.r378070

**Published:** 2025-04-28

**Authors:** Jodie Marquez

**Affiliations:** 1 The University of Newcastle, Callaghan, New South Wales, Australia

Thank you for the opportunity to review the response made by the authors.

 I appreciate that they have addressed my comments. One point remains outstanding. 

 The authors have clarified the aim of the study, to validate a tool to assess functional walking abilities - this has merit. However. the authors take this further to state: 

**in the abstract:** Better advice and training could reduce fall risk; 

**in the intro:** a promising tool to give specific advice and training in walker use and to reduce the risk of falling in people with dementia.

 The ability to educate and train this population to reduce falls needs validation from the literature. This is a statement that is far reaching in this population with cognitive/STM impairments that worsen with time.

Is the work clearly and accurately presented and does it cite the current literature?

Partly

If applicable, is the statistical analysis and its interpretation appropriate?

Yes

Are all the source data underlying the results available to ensure full reproducibility?

Yes

Is the study design appropriate and is the work technically sound?

Yes

Are the conclusions drawn adequately supported by the results?

Partly

Are sufficient details of methods and analysis provided to allow replication by others?

Yes

Reviewer Expertise:

healthy aging, stroke, rehabilitation, falls

I confirm that I have read this submission and believe that I have an appropriate level of expertise to confirm that it is of an acceptable scientific standard, however I have significant reservations, as outlined above.

# Reviewer report: Reviewer response for version 2

**DOI:** 10.5256/f1000research.180410.r378069

**Published:** 2025-04-26

**Authors:** Brittney Muir

**Affiliations:** 1 University of Washington, Seattle, USA

The authors have addressed my concerns. Thank you

Is the work clearly and accurately presented and does it cite the current literature?

Yes

If applicable, is the statistical analysis and its interpretation appropriate?

Yes

Are all the source data underlying the results available to ensure full reproducibility?

No source data required

Is the study design appropriate and is the work technically sound?

Yes

Are the conclusions drawn adequately supported by the results?

Yes

Are sufficient details of methods and analysis provided to allow replication by others?

Yes

Reviewer Expertise:

.

I confirm that I have read this submission and believe that I have an appropriate level of expertise to confirm that it is of an acceptable scientific standard.

# Reviewer report: Reviewer response for version 1

**DOI:** 10.5256/f1000research.145710.r351635

**Published:** 2025-01-29

**Authors:** Brittney Muir

**Affiliations:** 1 University of Washington, Seattle, USA

This article is a replication study of the work by Hunter *et al.* (2020). ^14^ However, the authors have translated the instrument into Dutch, and evaluated its use. The study found that  SUMAC-NL shows good to excellent reliability and content validity, and seems to be a promising tool to assess walking with a walker in people with dementia in the Netherlands.

 Major comments:

 I find the science to be sound, as it is by design a replication of previous work. I have major concerns about the value this study adds to science. While validating the tool in another language is an important step, there is little support on why this is novel publishable scientific work.

 Towards this, I am requesting the authors revise the introduction and discussion to explain why it is important to evaluate and publish this work on the translated device. Points that could be considered in this revision include: 1) Were you expecting the Dutch version to not be as valid/ reliable? 2) What value does this study bring other than having the tool in Dutch? Does it speak to the robustness of the original tool?

 Minor comments:

 I have no minor comments, I thought the paper was well-written, scientifically sound, and rigorous work was conducted and documented.

Is the work clearly and accurately presented and does it cite the current literature?

Yes

If applicable, is the statistical analysis and its interpretation appropriate?

Yes

Are all the source data underlying the results available to ensure full reproducibility?

No source data required

Is the study design appropriate and is the work technically sound?

Yes

Are the conclusions drawn adequately supported by the results?

Yes

Are sufficient details of methods and analysis provided to allow replication by others?

Yes

Reviewer Expertise:

aging, mobility, device evaluation and translation.

I confirm that I have read this submission and believe that I have an appropriate level of expertise to confirm that it is of an acceptable scientific standard, however I have significant reservations, as outlined above.

# Response

**Published:** 2025-02-25

**Authors:** Jesper Kroesen

**Affiliations:** Hanzehogeschool Groningen, The Netherlands

Dear Brittney, 

 Thank you for your valuable feedback. We will work on it and then upload a new version after that.

 Kind regards, Jesper Kroesen

# Response

**Published:** 2025-04-07

**Authors:** Jesper Kroesen

**Affiliations:** Hanzehogeschool Groningen, The Netherlands

Dear Brittney, 

*First of all, we would like to thank you for your effort in reviewing our study. Below is a response to the feedback and its incorporation.*

 Major comments:

 I find the science to be sound, as it is by design a replication of previous work. I have major concerns about the value this study adds to science. While validating the tool in another language is an important step, there is little support on why this is novel publishable scientific work.

 Towards this, I am requesting the authors revise the introduction and discussion to explain why it is important to evaluate and publish this work on the translated device. Points that could be considered in this revision include: 1) Were you expecting the Dutch version to not be as valid/ reliable? 2) What value does this study bring other than having the tool in Dutch? Does it speak to the robustness of the original tool?

***Author Response:** We adapted and  refined the **introduction** to make clear and substantiate the importance of translating the SUMAC into Dutch and why a reliability study is necessary after translation. This strengthened the scientific value of the study. In the **discussion**, we also addressed the robustness of the original SUMAC.*

 Minor comments:

 I have no minor comments, I thought the paper was well-written, scientifically sound, and rigorous work was conducted and documented.

***Author Response: **Thanks for the compliments and the effort taken to provide feedback on our study!*

# Reviewer report: Reviewer response for version 1

**DOI:** 10.5256/f1000research.145710.r270411

**Published:** 2024-05-10

**Authors:** Jodie Marquez

**Affiliations:** 1 The University of Newcastle, Callaghan, New South Wales, Australia

**Abstract:**

 The abstract needs to be revised to clearly outline the reason for the study and the primary aims. Concepts need to be introduced more clearly. For example:

  “the use of a walker in people with dementia is associated with a threefold increased odds of falls compared to their healthy peers”. This point is misleading – it suggests that walker use is associated with falls, yet it may be that if they didn’t use a walker the falls rate may be much higher. Please revise for clarity.  

 Please reconsider ‘Better advice and training could reduce falls risk – is this true in this population where short term memory is problematic? It would seem that the assessment tool would be used to warrant the prescription of the walker rather than to inform education. Please re consider the purpose of this tool. If this point is to be presented, please provide evidence in the introduction of evidence of the use of training /education to improve mobility in this population.

 The SUMAC was developed but why was the Dutch version required?

 Don’t use abbreviations not introduced eg PF domain, EQ

 Was intra-rater/ test-retest  reliability assessed?

**Introduction:**

 The background needs to describe what assessments are currently used to determine the need for a mobility aid, such as a walker, in the general population – and why these tools are not suitable for those with dementia Why is a dementia specific tool required?

 What makes the Dutch population any different to the global population – why is a Dutch specific tool required?

**Methods:**

 Not clear why a cognitive assessment score was not included in the demographic information –it would seem this information would be very important in determining what level of cognitive functioning is appropriate for clinical application of the tool

 In the ‘data collection’ section it states ‘ a convenience sample of five physiotherapist who were recruited’ else where it states “8 experts” please clarify.

 The expert panel are very homogenous with regard to age and experience  - this is a potential limitation to widespread adoption of the tool whereby reliability may differ for those outside of this age/experience range

**Discussion:**

 The authors conclude that the difference in reliability with the previous study may be due to the difference in the format in which the training was provided ( in person vs online) please clarify this. Was the content different? How would this affect the scoring?

 There were differences in findings but no recommendation to modify the tool for the Dutch population therefore the impact of the study is reduced

Is the work clearly and accurately presented and does it cite the current literature?

Partly

If applicable, is the statistical analysis and its interpretation appropriate?

Yes

Are all the source data underlying the results available to ensure full reproducibility?

Yes

Is the study design appropriate and is the work technically sound?

Yes

Are the conclusions drawn adequately supported by the results?

Partly

Are sufficient details of methods and analysis provided to allow replication by others?

Yes

Reviewer Expertise:

rehabilitation, ageing, exercise, physiotherapy

I confirm that I have read this submission and believe that I have an appropriate level of expertise to confirm that it is of an acceptable scientific standard, however I have significant reservations, as outlined above.

# Response

**Published:** 2024-05-31

**Authors:** Jesper Kroesen

**Affiliations:** Hanzehogeschool Groningen, The Netherlands

Dear Jodie, 

 Thank you for taking the time to review our study. We find your suggestions very valuable and will incorporate them into the article. On the advice of F1000research, we will do so after a second review by another researcher.

 Kind regards, 

 Jesper Kroesen

# Response

**Published:** 2025-04-07

**Authors:** Jesper Kroesen

**Affiliations:** Hanzehogeschool Groningen, The Netherlands

Dear Jodie, 

*First of all, we would like to thank you for your effort in reviewing our study. Below is a response to the feedback and its incorporation.*

**Abstract:**

 The abstract needs to be revised to clearly outline the reason for the study and the primary aims. Concepts need to be introduced more clearly. For example:

  “the use of a walker in people with dementia is associated with a threefold increased odds of falls compared to their healthy peers”. This point is misleading – it suggests that walker use is associated with falls, yet it may be that if they didn’t use a walker the falls rate may be much higher. Please revise for clarity.  

 Please reconsider ‘Better advice and training could reduce falls risk – is this true in this population where short term memory is problematic? It would seem that the assessment tool would be used to warrant the prescription of the walker rather than to inform education. Please re consider the purpose of this tool. If this point is to be presented, please provide evidence in the introduction of evidence of the use of training /education to improve mobility in this population.

 The SUMAC was developed but why was the Dutch version required?

 Don’t use abbreviations not introduced eg PF domain, EQ

 Was intra-rater/ test-retest  reliability assessed?

***Response** : We agree that the concept could be introduced more clearly. We have made a textual addition to a sentence in the **abstract** to clarify this. In addition, the abbreviations mentioned have been removed and replaced with full words. It was not mentioned in the abstract, but the discussion substantiated that implicit learning is still possible with the target audience.*

**Introduction:**

 The background needs to describe what assessments are currently used to determine the need for a mobility aid, such as a walker, in the general population – and why these tools are not suitable for those with dementia Why is a dementia specific tool required?

 What makes the Dutch population any different to the global population – why is a Dutch specific tool required?

***Response** :The **introduction** has been revised  and  refinedto more clearly justify  the necessity of translaing the SUMAC into Dutch and the rationale for subsequently conducting a reliability check on the translated SUMAC. We agree that this could be stated more clearly in the introduction*

**Methods:**

 Not clear why a cognitive assessment score was not included in the demographic information –it would seem this information would be very important in determining what level of cognitive functioning is appropriate for clinical application of the tool

 In the ‘data collection’ section it states ‘ a convenience sample of five physiotherapist who were recruited’ else where it states “8 experts” please clarify.

 The expert panel are very homogenous with regard to age and experience  - this is a potential limitation to widespread adoption of the tool whereby reliability may differ for those outside of this age/experience range

***Response** : We  recognize that the  absence of scores from a cognitive survey of people with dementia is an important oversight. Unfortunately, we  are unable to revisit participants as their cognitive state has changed since  3 years ago. Following the review, we have given this issue additional paid  attention  in the **discussion**.*

*Reading back, we understand that the distinction between the group of raters and the expert group for the validity assessment  was not clearly delineated. We  formulated this more clearly in the new version in the **methods section under: data collection**.*

*The limitations of a homogeneous expert group have also been incorporated into the **discussion***

**Discussion:**

 The authors conclude that the difference in reliability with the previous study may be due to the difference in the format in which the training was provided ( in person vs online) please clarify this. Was the content different? How would this affect the scoring?

 There were differences in findings but no recommendation to modify the tool for the Dutch population therefore the impact of the study is reduced

***Response** :We clarified the explanation in the difference in scores in the **discussion**.*
